# Shyness as an Anticipatory Form of Appeasement in Urban Black American Children

**DOI:** 10.3390/bs16101739

**Published:** 2026-09-24

**Authors:** Travis M. Wilson

**Affiliations:** Department of Psychology, Oberlin College, Oberlin, OH 44074, USA; twilson@oberlin.edu

**Keywords:** Black Americans, shyness, hedonic behavior, agonic behavior, care-eliciting behavior

## Abstract

This is the first empirical study to report on shyness in Black American children. Shyness is framed as serving the function of *appeasement*, that amid conditions of potential social conflict or power imbalance, the conciliatory nature of shyness engenders interpersonal cooperation to the benefit of the shy individual and the group to which he or she belongs. Peer nominations, teacher reports, and census data were used to explore age-related differences in the correlates of perceived shyness among 445 Black American students attending five urban elementary schools in the U.S. (grades 3–6; mean ages 9.4–12.3 years; 91% free or reduced lunch). Multilevel regression analysis yielded findings in at least partial support of four theoretically derived hypotheses. In partial support of the shyness as protection from environmental risk hypothesis (*H*_1_), Black girls (but not boys) and older students (grades 5 and 6) who resided in more disadvantaged neighborhoods were more frequently perceived by their peers as being shy. Consistent with the ascendance of shyness as appeasement hypothesis (*H*_2_), having low popularity was associated with higher levels of perceived shyness among students in grades 5 and 6, but not in grades 3 and 4. Consistent with the hedonic effects of shyness hypothesis (*H*_3_), among students in grades 5 and 6 (but not grades 3 and 4), being widely perceived as shy was associated with higher levels of prosocial behavior. Consistent with the care-eliciting effects of shyness hypothesis (*H*_4_), being widely perceived as shy was associated with having a closer relationship with the teacher, and this association was stronger among students in grades 5 and 6 than among students in grades 3 and 4. The study findings are discussed within a conceptual framework that integrates evolutionary, cultural, and ecological perspectives of human shyness.

## 1. Introduction

Since the publication of Charles Darwin’s *The Expression of the Emotions in Man and Animals* (Darwin, 1872/1998), thousands of scientific studies have reported on the nature, causes, and development of human shyness. Not one of these has focused explicitly on Black American children.^1^ Regrettably, this omission has contributed to limiting views of Black children’s vast socioemotional expression (Barbarin et al., 2019; Lozada et al., 2022) and to culturally limited understandings of the development of shyness itself (Yiu et al., 2020). The present study counters this trend by centering Black American children in the scientific discourse on human shyness.

We test the evolutionary claim that human shyness serves the function of *appeasement*, that amid conditions of potential social conflict or power imbalance, the conciliatory nature of shyness engenders interpersonal cooperation to the benefit of the shy individual and the group to which he or she belongs (Crozier, 2001; Keltner et al., 1997; Trower et al., 1999). Shyness may be especially likely to function as appeasement in Black American children. Given their racial minority status and chronic exposure to systemic racism, developing coping mechanisms that simultaneously protect the self against environmental threats and conserve the assets of their heritage culture is an essential developmental task for Black children (Spencer, 2006). Shyness may be one such mechanism. The behavioral repertoire that comes with being shy—quietness, deference, circumspection—may forestall social threats to Black children’s well-being and safeguard their membership to a valued peer group. This is a fundamental premise of our study.

We examine shyness among urban Black elementary school students in late childhood (ages 9–10) and early adolescence (ages 11–12). As children transition to adolescence, the salience of ethnic–racial identity (Umaña-Taylor et al., 2014), the intensity of self-consciousness (Harter, 2008), and the amount of time spent with peers (Savin-Williams & Berndt, 1990) are in continual ascendance, giving children ever more reasons to be shy and an ever-expanding social world in which to express their shyness. At the same time, the peer group itself is becoming more stable, more cohesive, and more influential (Laursen, 2018), providing a stronger foothold for the ongoing development of individual competencies and personal identity. For Black children especially, the peer group becomes a site of intensive racial socialization and a place to demonstrate one’s loyalty to the ethnic-racial ingroup (Wilson & Rodkin, 2013). It is within this cultural–developmental context that we frame the central question, *Does shyness emerge as an anticipatory form of appeasement among urban Black American children in the transition from late childhood to early adolescence?*

### 1.1. Integrating Evolutionary, Cultural, and Ecological Perspectives of Shyness

Shyness is a multisystemic phenomenon characterized as a behavioral state of inhibition often accompanied by physical symptoms like blushing, stammering, perspiring, or trembling; a mental state of intense self-consciousness; and feelings of inferiority, of being unwanted, or of intruding in a social space (Gunther et al., 2020; Lewinsky, 1941; Xu et al., 2020; Zimbardo, 1977). Shyness is a universal phenomenon (Gunther et al., 2020), though its expression and meaning vary between cultures (Yiu et al., 2020), between individuals within a culture (Zimbardo, 1977), and across settings within the same individual (Asendorpf, 1990). Reflecting the multisystemic nature and contextual variability of shyness, we integrate concepts from evolutionary, cultural, and ecological perspectives of shyness to frame our central study question.

#### 1.1.1. Evolutionary Perspectives of Shyness

In their broadest conceptualizations, evolutionary perspectives of shyness share the three-fold premise that (a) human shyness is a biologically prepared behavioral system, (b) a biologically prepared behavioral system can be viewed as an adaptation designed by natural selection to solve problems of survival and reproduction in ancestral environments, and (c) biologically prepared systems allow for cultural variation and flexibility to meet the specific demands of a local ecology (MacDonald, 2005; Stevenson-Hinde, 2000). Shyness can be viewed as a specified behavioral system that coordinates two broader behavioral systems: the defense system and the safety system (Stevenson-Hinde, 2000).

The defense system seeks out environmental danger and enables the individual to avoid threats to the self. The *agonic* sub-system, an evolved component of the defense system, enables humans to live close to each other in hierarchical social groups for the benefit of mutual defense and resource control (Trower et al., 1999). Agonic functioning involves verbal and non-verbal behaviors that signal one’s position in the social hierarchy, one’s understanding of the social hierarchy, or one’s intent to either challenge or submit to another group member’s social position. Dominance behaviors are used by higher-status individuals to signal their willingness to defend their elevated position. Appeasement behaviors are used by lower-status individuals to signal non-threat, conformity to group norms, and submissiveness to higher-status group members. As an *anticipatory* form of appeasement, shyness enables one to avoid interpersonal conflict and redirect one’s energies to cooperative interpersonal behaviors that form the basis of the safety system (Keltner et al., 1997).

The safety system, which includes the attachment and hedonic sub-systems, attends to stimuli that positively reinforce exploratory and affiliative behavior. Whereas the attachment sub-system consists of “vertically aligned” relationships between a dependent and its caregivers, the *hedonic* system consists of “horizontally aligned” relationships between mutually dependent individuals (Trower et al., 1999). When activated, both social sub-systems reduce defensive arousal and facilitate trusting interpersonal interactions. In secure attachment relationships, the dependent sends cues to the caregiver to elicit *nurturance*, searches for responsive nurturing signals from the caregiver, and positively reinforces the caregiver’s care provisions. In hedonic relationships, the individual sends *affiliative* cues to their fellow group members to elicit social connection, searches for reassurance signals from their group members, and positively reinforces these reciprocating behaviors.

The shyness behavioral system can thus be viewed as a subtle means of coordinating the agonic and hedonic systems. Shy children are prone to averting their gaze, smiling, touching their face (a sign of embarrassment), using nonintrusive speech styles (e.g., nods of the head, passive agreement) and using constrained gestural expression (Asendorpf, 1990; Keltner et al., 1997). Some of these behaviors are clear signs of appeasement (e.g., gaze aversion) that, having demonstrated acquiescence to the group hierarchy, *in turn* enable the shy child to send reassurance signals to peers and care-eliciting signals to adults. Other shy behaviors (e.g., smiling) signal appeasement and an orientation toward affiliation *simultaneously*. With repeated practice across a wide range of social environments, the shy individual entrains a sophisticated behavioral repertoire that nimbly avoids social conflict, reinforces peer affiliation, and elicits care from trusted adults. Just as important, the shy individual gains a *reputation* among peers (Chen, 2010) that they are aligned with the social hierarchy and invested in contributing to cohesive group functioning. The shy individual becomes an asset to the group.

Several indicators suggest that children may become better adept at coordinating the agonic and hedonic systems as they transition into early adolescence. As peer group hierarchies become clearer and more stable, children increasingly prioritize popularity over other social goals (Lafontana & Cillessen, 2010). They rely more on peers for social comparison and emotional support, become more selective in who they pay attention to, and become savvier in how they inhibit their behavior with peers (Del Giudice, 2014; Hay & Ashman, 2003). Children also evidence more frequent prosocial behavior inearly adolescence, due to their enhanced abilities to understand multiple perspectives, to infer how others feel, and to perceive opportunities to help or comfort others (Eisenberg et al., 2015). These prosocial behaviors are not merely “altruistic” but often instrumental and egoistic in nature, as they help establish interpersonal trust and foster alliances for future aid (Hawley, 2014). Working in synchrony, the acquired attentional, motivational, and behavioral resources may enable early adolescents to strategically enact appeasement as a means to preserve their personal safety and group membership.

To summarize, evolutionary perspectives emphasize that human shyness is a multidimensional, biologically prepared, and universal behavioral system. As an evolved system, the function of shyness in adults is anticipatory appeasement, which is the ability to mitigate within-group threats to the individual and redirect one’s energies to elicit care from adults or to elicit affiliative, prosocial interactions with fellow group members. What remains unclear is the developmental timing of the emergence of appeasement as a primary function of shyness. However, the normative social, cognitive, and behavioral changes that occur from roughly 7 to 12 years of age suggest that shyness is increasingly likely to function as appeasement during the transition from childhood to early adolescence.

#### 1.1.2. Cultural Perspectives of Shyness

As a biologically prepared system, there is cultural variation in how shyness is expressed to meet the specific demands of a local ecology. Thus, there is the possibility that appeasement may be a *normative* function of shyness but not *universal* across all cultures. To conceptualize how shyness may function in Black American children, we draw upon two prevailing cultural perspectives: the Multidimensional Model of Shyness (MMS; Xu et al., 2009; Xu et al., 2020) and the Contextual-Developmental Model of shyness (CDM; Chen et al., 2006). The MMS asserts that shyness functions as appeasement universally across cultures. The CDM does not explicitly address *why* some children act shy (i.e., the function of shyness) but instead addresses *how* shy children in different cultures are perceived and treated by their peers (i.e., adjustment outcomes); nonetheless, the CDM allows for the possibility that shyness functions as appeasement universally across cultures.

The Multidimensional Model of Shyness posits that shyness universally functions as appeasement in cultures across the individualistic–collectivistic spectrum (Xu et al., 2009; Xu et al., 2020). According to this view, three distinct forms of shyness appear cross-culturally, albeit to varying degrees: shyness toward strangers (fear of novelty), anxious shyness (fear of social disapproval), and regulated shyness (fear of being the center of attention). All three forms are present before early adolescence and are characterized by relatively infrequent social interaction, reticence, and modest behavior, but vary in their emotional experience and eliciting conditions (Xu et al., 2020). Due in part to individual differences in effortful control (Xu et al., 2009), the three forms of shyness also show different patterns of socioemotional adjustment, with regulated shyness typically faring better than the other two forms. Despite these differences, MMS exponents argue that all three forms “serve a similar adaptive function of appeasement that is essential for establishing and/or reestablishing cooperative social relations” and “are often perceived by others as a promise to fit in and to engage in appropriate behavior worthy of others’ trust and respect” (Xu et al., 2020, p. 227). It is the quieting of behavior that placates others, regardless of the underlying fear or the cultural context. Within the MMS framework, then, shyness is expected to function as appeasement in Black American children.

The Contextual–Developmental Model of shyness posits that peer social interaction processes mediate the influence of culture on the individual adjustment of shy children. CDM exponents make three assertions: (1) In individualistic-oriented cultures, autonomy is a deeply valued developmental goal, self-assertion is viewed as a marker of social competence, and shyness is viewed by one’s peers as socially immature. (2) In communalistic-oriented cultures, group harmony is a deeply valued developmental goal, self-restraint is viewed as a marker of social competence, and shyness is viewed by one’s peers as socially mature. (3) Correspondingly, shy children in communalistic cultures have better adjustment outcomes (e.g., having more friends, being well-accepted by classmates) than do shy children in more individualistic cultures (Chen, 2010). A sizeable empirical record substantiates these assertions. Shy youth in communalistic cultures such as China, Japan, and Korea tend to have satisfying friendships, perform well academically, and are well liked by classmates (e.g., Chen et al., 1992, 2006; Heinrichs et al., 2006; West & Newman, 2007), whereas shy youth in individualistic cultures such as Australia, Canada, and the United States tend to have few friends, struggle academically, and are prone to peer rejection (e.g., Chen et al., 1997; Coplan et al., 2013; Heinrichs et al., 2006; Rapee et al., 2011). Interestingly, shy youth in “culturally blended” settings, such as children of Asian immigrants in Canada, tend to have a mixed profile of positive and negative social adjustment, presumably reflecting a blend of communalistic messaging at home and individualistic messaging from mainstream society (Chen & Tse, 2008). The latter finding is of relevance to Black American children, who as cultural minorities similarly experience both communalistic and individualistic messaging in their daily lives. Communalism is an essential aspect of Black American culture (Boykin et al., 1997; DuBois, 1903; Jagers & Mock, 1995), and espousing communalism is integral to Black youths’ thriving (Gooden & McMahon, 2016). Within the CDM framework, then, with its primary emphasis on the individualism–communalism dimension of culture, Black American children may be regarded as functioning within a blended cultural niche as they navigate the individualistic values inherent in dominant mainstream society along with the communalistic values inherent in their heritage communities. According to this logic, shy Black children can be expected to have a mixed profile of social adjustment, being neither widely liked nor disliked by peers. However, Black children face additional social challenges that are not accounted for by the CDM. In addition to navigating the sometimes conflicting values of the dominant culture and the priorities of their heritage culture, Black children must also navigate dynamics associated with being a member of a historically oppressed group, a dynamic known as the triple quandary (Boykin, 1986).

To summarize, the MMS and CDM frameworks emphasize two different aspects of shyness: the underlying and cross-culturally consistent *function* of shyness (MMS), and the culturally variant subjective *meaning* of shyness (CDM). With their respective focuses on function and meaning, the two frameworks are not contradictory but complementary. The MMS posits that the quieting, respectful, appeasement-like behaviors of shyness mitigate social conflict and provide fertile grounds for establishing cooperative relations; this is expected for any cultural group across the individualism–communalism spectrum, including Black American children. The CDM posits that the subjective meaning of shy behavior varies across cultures along the individualism–collectivism spectrum: children in communalistic cultures are expected to evidence positive social adjustment; children in individualistic cultures are expected to evidence negative social adjustment. The CDM anticipates that shy Black children may have a “mixed” social adjustment profile (liked by some peers and avoided by others) due to their navigating the sometimes conflicting values of mainstream society and Black cultural heritage. However, the CDM does not explicitly account for the triple quandary that Black children face in navigating majority and minority cultural influences along with their historically marginalized status. A comprehensive understanding of Black children’s shyness must account for their exposure to diverse cultural messaging as well as their continual exposure to systemic racism.

#### 1.1.3. Ecological Perspectives of Shyness

The Phenomenological Variant of Ecological Systems Theory (PVEST; Spencer, 2006) is a widely adopted framework for theorizing about Black children’s socioemotional development because of its thorough analysis of ecological conditions (e.g., poverty) and minority cultures (Barbarin et al., 2019).

A core principle of the PVEST is a recognition that structural racism is a normative and pervasive experience for Black children and their families. Due to a long history of racially discriminatory policies in the U.S., Black Americans have elevated odds of living in single-parent households and in neighborhoods characterized by economic disadvantage, unemployment, crime, and violence (Barbarin et al., 2019; Sharkey & Sampson, 2015). Experimental studies (Sanbonmatsu et al., 2006; Kling et al., 2007) and correlational data (Kohen et al., 2008; Snedker & Herting, 2016) indicate that living in disadvantaged neighborhoods puts youth at elevated risk of disease morbidity, anxiety, depression, and maladaptive behavior, and that the insidious effects of these risks accrete over the course of an individual’s development (Barbarin et al., 2019). Whether these patterns vary by gender is unclear. Some correlational data indicate that neighborhood disadvantage and socioemotional distress covary more strongly for boys than for girls (Leventhal & Brooks-Gunn, 2011), whereas other data evidence no such gender difference (Mistry et al., 2002). Based on these findings, we conjecture that Black children who reside in more disadvantaged neighborhoods are more likely to adopt a shy behavioral orientation as a whole-body response to the presence of environmental threats or perceived lack of safety. This question has not been studied to date.

A second core PVEST principle is that the most proximal layers of the social ecology are especially powerful forces in shaping youths’ coping strategies. The strongest sources of influence are parent–child and sibling relationships in the early years of life but later include a wider range of people as children spend more time outside the home. Upon school entry, the peer group and student–teacher relationships have an outsized impact on children’s social development. In school contexts, children’s agonic functioning is commonly indexed by their levels of *perceived popularity*, a measure of power, prestige, and influence in the peer ecology (Cillessen & Rose, 2005); hedonic functioning by their level of *prosocial* behavior and *likeability* among peers (McDonald & Asher, 2018); and attachment-like functioning by the *closeness* of their relationships with teachers (Saft & Pianta, 2001).

Although prioritizing status (popularity) increasingly with age is a normative pattern found across many ethnic–racial groups, it may have particular significance for Black youth given their minoritized status in the U.S. Some research indicates that Black youth in racially diverse classrooms attain more elevated levels of popularity than children of other racial groups, suggesting that Black children’s status strivings in the peer ecology serve to counteract their marginalized status in the broader society (Rodkin et al., 2000; Graham & Juvonen, 2002). Other research indicates that Black children who have more racially segregated friendships and peer groups are perceived as more popular by their classmates (whereas the opposite is true for White students), suggesting that Black children are rewarded with social prestige when they demonstrate loyalty to the ingroup (Wilson & Rodkin, 2011, 2013). Together, these findings attest to the value that Black children place on belonging to the ingroup and having solidarity with same-race peers. For less popular Black children, who may feel vulnerable to being negatively judged or rejected by their ingroup peers, shyness may be instrumental in protecting their group membership and eliciting care from teachers. This may be especially true for older Black children given the increasing salience of popularity with age.

A key to understanding prosocial behavior in middle childhood and adolescence is its instrumental nature (Del Giudice, 2014). Prosocial behavior is not only used for altruistic purposes but also to vie for new friendships, to bid entry into games and play situations, and to attain higher status (Hawley & Bower, 2018). Research indicates that Black children who are perceived as more prosocial are better liked by their classmates (of all races), less likely to suffer peer rejection, and have higher status in the peer group (Wilson & Rodkin, 2013). Still, as with children of other ethnic–racial groups, the most popular Black children use a balance of prosocial and coercive strategies (e.g., teasing, spreading rumors) to attain and secure their social position (Graham & Echols, 2018). For shy children who wish to avoid standing out in social settings, stricter reliance on subtle prosocial interactions is the safer strategy. Mainstream research indicates that shy children’s prosocial behavior with peers is often covert, as they are more likely to be prosocial in response to requests (vs. spontaneously) and in one-on-one (vs. small or large group) social settings (Eisenberg et al., 2015). This behavioral pattern may partially explain why shy children in communalistic cultures, with their valuing of self-effacement, are more widely liked by their peers than are their shy counterparts in more individualistic-oriented cultures (Chen, 2010). Whether shy Black children are more similar in this respect to shy children in communalistic or individualistic cultures is an unexplored question.

The importance of teacher–child relationships on children’s development also has been well documented. Students who experience positive student–teacher relationships marked by warm interactions and open communication tend to feel that their teachers care about them and have better long-term academic outcomes (Bakadorova & Raufelder, 2018; Burchinal et al., 2002; Hamre & Pianta, 2005). There is reason to believe that Black students may be culturally oriented to seek out positive relationships with their teachers in ways different than their majority peers. As a response to historical and contemporary racial inequities in home ownership, health care, and mass incarceration, Black families have long developed extended social networks that include biological family members as well as “fictive kin” and natural mentors to meet their families’ needs (Stern et al., 2023). Early in life, Black children’s fictive kin and natural mentors may be limited to select childcare providers and other adults trusted by their parents. With age, as Black children develop greater autonomy outside of the home, they may seek out teachers, coaches, and other adults in their community as natural mentors. For shy Black children who have relatively smaller social networks, the natural mentorship of caring teachers may provide a much-needed safe haven.

## 2. The Present Study

With a community sample of urban Black American elementary school students, we test the evolutionary claim that human shyness serves the function of anticipatory appeasement, promotes affiliative behaviors with peers, and engenders close student–teacher relationships (Keltner et al., 1997). We focused on students in late childhood and early adolescence (grades 3–6, ages 9 to 12), which represents an important period in the development of shyness, peer socialization, and social competence with peers. Compared to early childhood, in late childhood and early adolescence the peer group is more hierarchically structured, shyness is more stable as observed by peers, and individuals use prosocial behavior more strategically to form social alliances (Crozier, 2000; Del Giudice, 2014; Karevold et al., 2012).

The literature reviewed gives rise to four developmental hypotheses. *Shyness as protection from environmental risk (Hypothesis 1)*: residing in more disadvantaged neighborhoods will be associated with higher levels of perceived shyness for children in all four grade levels. *Shyness as anticipatory appeasement (Hypothesis 2)*: having low popularity will be associated with higher levels of perceived shyness; reflecting the higher levels of self-consciousness and peer group hierarchy in early adolescence (vs. childhood), this association will be stronger in the upper grades (5 and 6) than in the lower grades (3 and 4). *Hedonic effects of shyness (Hypothesis 3)*: being widely perceived as shy will be associated with higher levels of prosocial behavior and likeability among peers; congruent with the hypothesized age-related differences in shyness-as-appeasement, this association will be stronger in the upper grades than in the lower grades. *Care-eliciting effects of shyness (Hypothesis 4)*: being widely perceived as shy will be associated with having a closer relationship with the teacher; congruent with the hypothesized age-related differences in shyness-as-appeasement, this association will be stronger in the upper grades than in the lower grades.

To test the study hypotheses comprehensively, and to mitigate the problem of shared method variance, we use information from classmates, teachers, and demographic records (census information). We adopt a widely used method that privileges understanding how shyness functions among children in school contexts, whereby classmates evaluate each other on shyness and other characteristics (i.e., peer nominations; Chen, 2010). To test *Hypothesis 1*, we use a measure of neighborhood socioeconomic disadvantage derived from U.S. census data. To test *Hypotheses 2–4*, we use peer nominations of children’s shyness, popularity, prosocial behavior, likeability, and student–teacher closeness; to assess the robustness of findings, we also include teacher reports of children’s prosocial behavior and student–teacher closeness (the study did not include teacher reports on student shyness, popularity, or likeability).

## 3. Method

### 3.1. Participants

This study is part of a larger investigation of the social adjustment of low-income, urban ethnic minority children and young adolescents. The study targeted all students in grades 3–6 in five schools in a large city in the Great Lakes region of the United States. There was one classroom per grade level at each school, resulting in a balanced design of five classrooms per grade level in the study. The present report considers Black students exclusively in these 20 classrooms. The five schools were similar in grade-level structure and demographics. All five schools housed grades k-8 and were predominantly low-income (free or reduced lunch status: *M* = 91%) and Black (*M* = 87%). The majority of students attending these schools lived in low-income neighborhoods; according to U.S. census neighborhood block-level information (U.S. Census Bureau, n.d.), the mean unemployment rate was 14.1% (*SD* = 6.3%) and the mean per capita income was $15,200 (*SD* = $6100). The study included a survey assessment in the fall and a second survey assessment in the spring. The present report focuses exclusively on the spring assessment (mid-April), as the shyness measure was not included in the fall.

The following consent procedure was approved by the Oberlin College Institutional Review Board. Consent letters describing the study were sent by first-class mail to the parents of children in participating classrooms. An identical copy of the consent letter was distributed to each student to take home to his or her parents two weeks before data collection. Parents were informed that if they did not want their child to participate in the study, they were either to return the consent letter to the teacher or to call the school, their child’s teacher, or the principal investigator at the number provided on the letter. Study participation also required written student assent.

Participant characteristics are presented in Table 1. Among the 559 eligible Black students, 445 (79.6%) participated in the spring survey, including 204 boys and 241 girls. The participation rate did not differ significantly by sex, *χ*^2^_(1)_ = 1.68, *p* = 0.20, but differed significantly by grade level (range: 73.3% to 88.3%), *χ*^2^_(3)_ = 10.9, *p* < 0.05. Participant gender distribution also varied by grade level, *χ*^2^_(3)_ = 9.37, *p* < 0.05; girls were overrepresented in grades 4–6 but underrepresented in grade 3. However, when the participants were analyzed as two cohorts (grades 3–4 combined vs. grades 5–6 combined), the gender distribution did not vary by cohort, *χ*^2^_(3)_ = 0.14, *p* = 0.71.

### 3.2. Procedure

Study measures were obtained from student questionnaires and teacher questionnaires. Students completed surveys in a single 40 min session in their respective classrooms. One member of the research team read aloud each questionnaire item, as two or three undergraduate research assistants monitored and assisted students individually, as needed. The order of student questionnaire measures was randomized for each classroom. Teachers completed a questionnaire on each of their participating students within one week of the classroom survey.

### 3.3. Measures

In this section, detailed information on all study measures and their original scoring is provided. Information on how original measures were transformed prior to analysis is provided in the *Analysis Plan* section (Section 3.4) that follows.

#### 3.3.1. Demographic Information

***Gender***: The teacher reported the gender of each participating child. Gender was coded as 0 = boy, 1 = girl, as no gender identities apart from boy or girl were reported. Responses to this measure may reflect a binary conceptualization of gender and in some cases may not accurately represent how the individual child self-identifies.

***Neighborhood Socioeconomic Disadvantage (NSD)***: U.S. census data were used to obtain three indicators of neighborhood block-level socioeconomic information for each participating student’s residential neighborhood: per capita income, median household income, and percent unemployment (U.S. Census Bureau, n.d.). Neighborhood per capita income ranged from $3600 to $44,100, with a median of $14,200. Median household income ranged from $6900 to $84,800, with a median of $25,500. Percent unemployment ranged from 1.1% to 32.7%, with a median of 12.9%. Each index was standardized; percent unemployment was then multiplied by −1 to attain similar valence across the three indices. A composite neighborhood socioeconomic disadvantage (NSD) score was computed for each child by (1) calculating the mean of the three standardized indexes; (2) standardizing the mean score; and then (3) multiplying the standardized mean score by −1. NSD scores had a mean of 0 and a standard deviation of 1, where positive scores indicated above-average disadvantage and negative scores indicated below-average disadvantage.

#### 3.3.2. Teacher Questionnaire

Teachers completed the Interpersonal Competence Scale—Teacher (ICS-T; Cairns et al., 1995) on each of their participating students. The present investigation used the Prosocial Behavior subscale, which is composed of four 5-point Likert items. Teachers also completed the Student–Teacher Relationship Scale (STRS; Pianta, 2001) on each of their participating students. The present investigation used the Student–Teacher Closeness subscale, which measures a teacher’s perceptions of how a child feels about their relationship; this subscale is composed of four 5-point Likert items.

***Prosocial Behavior***: The Prosocial Behavior subscale was the mean of “cheers up others,” “helpful to others,” “kind to others,” and “friendly”; α = 0.84.

***Student–Teacher Closeness***: The Student–Teacher Closeness subscale with the teacher scale was the mean of “trusts me,” “feels that I respect her/him,” “avoids contact with me” (reverse coded), and “feels that I treat her/him unfairly” (reverse coded); α = 0.83.

#### 3.3.3. Peer Nominations

We used a peer nominations procedure similar to prior research on children’s shyness (Yang et al., 2015). There were six peer nomination items and one per questionnaire page. Students reported on classmates’ shyness, prosocial behavior, social status among peers (i.e., perceived popularity), likeability, and relationship with the teacher. Below each item was a classroom roster with the name of each student listed alphabetically; nonparticipants were listed on rosters to maintain the anonymity of the study participants. The participants were asked to circle the names of students who they felt best fit the item description (e.g., “These kids are really SHY”). For ecological validity, the participants were allowed to circle an unlimited number of classmates’ names, but self-nominations were excluded from analysis. A proportion score was calculated for each item as the quotient of nominations received for the given item divided by the number of nominators in the classroom. The large number of respondents, the unlimited number of nominations permitted for each item, and the face validity of items ensure robust measurement properties even for single-item peer nomination measures. This measurement procedure is consistent with recommended practice (Cillessen & Bukowski, 2018).

***Perceived Shyness***: Participating students were asked to circle the names of students who were shy (i.e., “These kids are really SHY. These kids act shy around other kids or adults”). This measure of perceived shyness was a modified adaptation of a peer nomination measure used in prior research (Yang et al., 2015). The peer nomination method for identifying shy children is posited to have sound cultural validity because it draws on the perspectives of classmates who together co-construct the meaning of shyness in a given cultural milieu (Chen, 2010).

***Prosocial Behavior***: Prosocial behavior was measured as the mean of two items (α = 0.87): cooperative (i.e., “These kids COOPERATE. Here are kids who really cooperate—they pitch in, share, and give everyone a turn”) and nice (i.e., “These kids are always willing to do something NICE for somebody else, and are really nice people”). This measure has strong convergent validity with parent- and teacher-reported constructive social skills among elementary school children (Eisenberg et al., 1996).

***Perceived Popularity***: One item was used to measure each child’s *perceived popularity* among peers (i.e., “These are the most POPULAR kids in my class”; see Rodkin et al., 2000).

***Likeability***: One item was used to measure children’s *likeability* among peers (i.e., “These are the kids whom I would LIKE MOST to play with”; see Zhang et al., 2018).

***Gets Along with the Teacher***: One item was used to measure how well each child gets along with the teacher (i.e., “These children GET ALONG WELL WITH THEIR TEACHERS. They like to talk to their teachers, and their teachers enjoy spending time with them”). This measure has been validated among elementary school students (Hughes & Kwok, 2007).

### 3.4. Analysis Plan

Multilevel modeling was used to test the study hypotheses. Each model had a two-level hierarchical structure, with children nested within classrooms, and a random intercept to allow for unexplained mean classroom-level differences. Independent variables were standardized prior to analysis to mitigate multicollinearity; shyness was log-transformed before standardization to correct for skewness. For parsimony, grade level was operationalized dichotomously: a younger cohort (grades 3–4) versus an older cohort (grades 5–6). Gender and grade cohort were entered in models as main effects and in two-way interactions with predictors of interest. Post hoc probing of significant interactions was conducted per recommended practice; online computational tools were used to calculate simple slopes (Preacher et al., 2006).

## 4. Results

Bivariate correlations and descriptive statistics for study variables are presented in Table 2. Shyness was moderately but significantly correlated with all peer nomination and teacher-reported measures. The presentation of the results addresses the study hypotheses sequentially and proceeds in three sections: (a) the eliciting conditions of shyness, (b) the hedonic effects of shyness, and (c) the care-eliciting effects of shyness.

### 4.1. Eliciting Conditions of Shyness

The first analysis permitted testing of the two study hypotheses concerning the *eliciting conditions* of shyness: (1) *Shyness as protection from environmental risk* (*H*_1_): residing in disadvantaged neighborhoods is associated with higher levels of perceived shyness for children in all four grade levels; (2) *Shyness as anticipatory appeasement* (*H*_2_): having low popularity is associated with higher levels of perceived shyness, and more strongly so in the upper grades than in the lower grades (*H*_2_). To test these hypotheses, we fit two linear mixed-effects models in SPSS 32.0 with restricted maximum likelihood (REML) estimation. Each model predicted perceived shyness (proportion score, Level 1) nested within classrooms (Level 2). Random intercepts were modeled for classrooms. In Model 1, gender, cohort (grades 3–4 vs. grades 5–6), neighborhood disadvantage (ND), and perceived popularity were entered as main effects. In Model 2, all two-way interactions between predictors were entered. The results provided at least partial support for both hypotheses.

#### 4.1.1. Gender Differences in Levels of Shyness

In Model 1, the fixed effect of gender was statistically significant, *b* = 0.026, SE = 0.005, 95 CI [0.015, 0.037], *p* < 0.001, indicating higher shyness scores for girls (standardized *M* = 0.17, *SD* = 1.09) than for boys (standardized *M* = −0.20, *SD* = 0.85). In Model 2, the gender × cohort interaction did not reach significance, indicating that the gender difference in perceived shyness was consistent across the younger and older grade cohorts.

#### 4.1.2. Neighborhood Disadvantage

The results provided partial support for *H*_1_. In Model 1, the association between neighborhood disadvantage and shyness was marginally significant, *b* = 0.010, SE = 0.006, 95 CI [−0.001, 0.021], *p* = 0.082. In Model 2, the gender x neighborhood disadvantage interaction was significant, *b* = 0.010, SE = 0.005, 95 CI [0.0001, 0.021], *p* = 0.048, indicating that this association differed between girls and boys. Girls with higher levels of neighborhood disadvantage had higher levels of shyness, *b* = 0.019, SE = 0.0073, *p* = 0.010; for boys, the association was null, *b* = −0.0021, SE = 0.0082, *p* = 0.79. The cohort x neighborhood disadvantage also was significant, *b* = 0.014, SE = 0.006, 95 CI [0.003, 0.025], *p* = 0.015, indicating that this association differed between younger and older students. In the older cohort, students with higher levels of neighborhood disadvantage had higher levels of shyness, *b* = 0.022, SE = 0.0056, *p* < 0.001; in the younger cohort, the association was null, *b* = −0.0055, SE = 0.0061, *p* = 0.36. Thus, the results provided support for *H*_1_ among girls and among older students.

#### 4.1.3. Perceived Popularity

The results provided full support for *H*_2_. In Model 1, the fixed effect of popularity was significant, *b* = −0.028, SE = 0.005, 95 CI [−0.038, −0.018], *p* < 0.001, indicating higher shyness scores for students with lower popularity. In Model 2, the cohort x popularity interaction was significant, *b* = −0.016, SE = 0.005, 95 CI [−0.026, −0.006], *p* = 0.002, indicating that the popularity–shyness association differed between younger and older students. In the older cohort, students with lower levels of popularity had higher levels of shyness, *b* = −0.042, SE = 0.0070, *p* < 0.001; in the younger cohort, the association was null, *b* = −0.010, SE = 0.0078, *p* = 0.19. The gender × popularity interaction did not reach significance, indicating that the association did not differ between girls and boys.

#### 4.1.4. Summary

To summarize, girls (not boys) and students in the older (not younger) cohort who resided in more disadvantaged neighborhoods were more likely to be perceived by peers as being shy, as were older (not younger) children who had lower levels of popularity in the peer group. These results are consistent with the notion that some urban Black American children acquire a general disposition to be shy as an anticipatory form of appeasement to mitigate harm from potential social threats in their distal (neighborhood) and proximal (peer group) social environments. Whether the disposition to be shy in turn begets cooperative and affiliative behaviors with peers—the *hedonic* effects of shyness and appeasement—was addressed in the next analysis.

### 4.2. The Hedonic Effects of Shyness

The second analysis permitted testing of the *hedonic effects of shyness hypothesis (H*_3_*)*: being widely perceived as shy is associated with higher levels of prosocial behavior and likeability among peers, and more strongly so among students in the upper grades than in the lower grades. Two multilevel models were fit for each of three dependent variables: peer-nominated prosocial behavior, teacher-rated prosocial behavior, and peer-nominated likeability. In Model 1, gender, cohort, neighborhood disadvantage (ND), perceived popularity, and perceived shyness were entered as predictors. Perceived shyness was the focal predictor; the other predictors were entered as controls to isolate the unique association between shyness and each dependent variable above and beyond factors that elicit shyness itself. In Model 2, the gender x shyness and cohort x shyness interactions were entered to examine whether the predictive effect of shyness differed by gender or by cohort. The model details are provided in Table 3 and summarized below.

#### 4.2.1. Prosocial Behavior

The analysis of *peer-nominated* prosocial behavior provided robust support for *H*_3_. In Model 1, the fixed effect of perceived shyness was significant, *b* = 0.023, SE = 0.006, 95 CI [0.010, 0.035], *p* < 0.001, indicating higher peer-nominated prosocial scores for students with higher perceived shyness scores. In Model 2, the cohort × shyness interaction was significant, *b* = 0.019, SE = 0.006, 95 CI [0.007, 0.031], *p* = 0.002, indicating that the shyness–prosocial association differed between younger and older students. In the older cohort, students with higher levels of shyness had higher peer-nominated prosocial scores, *b* = 0.041, SE = 0.0086, *p* < 0.001; in the younger cohort, the association was null, *b* = 0.0034, SE = 0.0090, *p* = 0.70. The gender x shyness interaction did not reach significance, indicating that the association did not differ between girls and boys.

The analysis of *teacher-rated* prosocial behavior also provided support for *H*_3_. In Model 1, the fixed effect of perceived shyness was not significant, *b* = 0.048, SE = 0.043, 95 CI [−0.037, 0.134], *p* = 0.27. However, in Model 2, the cohort x shyness interaction was significant, *b* = 0.11, SE = 0.042, 95 CI [0.032, 0.195], *p* = 0.007, indicating that the shyness–prosocial association differed between younger and older students. In the older cohort, students with higher levels of shyness had higher levels of teacher-reported prosocial behavior, *b* = 0.18, SE = 0.060, *p* = 0.004; in the younger cohort, the association was null, *b* = −0.051, SE = 0.062, *p* = 0.40.

#### 4.2.2. Likeability

The analysis of peer-nominated likeability did not yield support for *H*_3_. In Model 1, the fixed effect of perceived shyness was not significant, *b* = 0.001, SE = 0.005, 95 CI [−0.009, 0.011], *p* = 0.45. In Model 2, the cohort x shyness interaction was not significant, contrary to *H*_3_. The gender x shyness interaction also was not significant.

#### 4.2.3. Summary

To summarize, the results largely substantiated the study hypotheses regarding the hedonic effects of shyness. Among the older cohort (but not the younger cohort), students who were more frequently perceived by peers as being shy were also more frequently perceived by peers and by teachers as being prosocial. The association between perceived shyness and likeability among peers was null for both cohorts, indicating that shy children are neither more nor less liked than their non-shy peers. These results are consistent with the notion that for urban Black Americans, the *hedonic effects* of shyness are more normative in early adolescence than in childhood. Whether the *care-eliciting* effects of shyness follow a similar pattern was addressed in the final analysis.

### 4.3. The Care-Eliciting Effects of Shyness

The final analysis permitted testing of the *care-eliciting effects of shyness hypothesis (H*_4_*)*: being widely perceived as shy is associated with having a closer relationship with the teacher, and more strongly so among students in the upper grades than in the lower grades. To test this hypothesis, two multilevel models were fit for each of two dependent variables: peer-nominated *gets along with the teacher* and teacher-rated *student–teacher closeness*. In Model 1, gender, cohort, neighborhood disadvantage (ND), perceived popularity, and perceived shyness were entered as predictors. Perceived shyness was the focal predictor; the other predictors were entered as controls to isolate the unique association between shyness and each dependent variable above and beyond factors that elicit shyness itself. In Model 2, the gender × shyness and cohort × shyness interactions were entered to examine whether the predictive effect of shyness differed by gender or by cohort. The regression model details are provided in Table 4 and summarized below. The results provide substantial support for *H*_4_.

#### 4.3.1. Peers’ Perceptions

The analysis of the peer-nominated *gets along with the teacher* model results provided robust support for *H*_4_. In Model 1, the fixed effect of perceived shyness was significant, *b* = 0.041, SE = 0.007, 95 CI [0.027, 0.055], *p* < 0.001, indicating higher levels of the gets along with the teacher item for students with higher levels of perceived shyness. In Model 2, the cohort x shyness interaction was significant, *b* = 0.017, SE = 0.007, 95 CI [0.003, 0.031], *p* = 0.015, indicating that this association differed between younger and older students. The association was positive in both cohorts, albeit stronger in the older cohort, *b* = 0.058, SE = 0.010, *p* < 0.001, than in the younger cohort, *b* = 0.025, SE = 0.011, *p* = 0.02.

#### 4.3.2. Teachers’ Perceptions

The analysis of teacher-rated *student–teacher closeness* provided partial support for *H*_4_. In Model 1, the fixed effect of perceived shyness was significant, *b* = 0.099, SE = 0.037, 95 CI [0.026, 0.172], *p* = 0.008, indicating higher levels of student–teacher closeness for students with higher levels of perceived shyness. In Model 2, the cohort x shyness interaction was not significant, contrary to *H*_4_. However, the gender × shyness interaction was significant, *b* = −0.080, SE = 0.039, 95 CI [−0.156, −0.004], *p* = 0.039, indicating that the association differed by gender. Boys with higher levels of shyness had higher levels of student–teacher closeness, *b* = 0.21, SE = 0.063, *p* = 0.001; among girls, the association was null, *b* = 0.046, SE = 0.046, *p* = 0.32.

#### 4.3.3. Summary

To summarize, the results largely substantiated the study hypothesis concerning the care-eliciting effects of shyness. In both the younger and older cohorts, higher levels of shyness were linked with closer student–teacher relationships. From the perspective of peers (but not teachers), this association was stronger in the older cohort than in the younger cohort. From the perspective of teachers (but not peers), this association was stronger among boys than among girls. These results suggest that for urban Black American students, the *care-eliciting effects* of shyness are apparent in late childhood and may be more pronounced among boys and in early adolescence.

## 5. Discussion

In the first study to examine shyness in Black American children, we tested the evolutionary claim that human shyness is an anticipatory form of appeasement, which is the process by which individuals placate or submit to others in situations of potential or actual conflict (Crozier, 2001; Keltner et al., 1997; Xu et al., 2020). Appeasement begins when the conditions of the social milieu lead an individual to anticipate derision or hostility from others; it is expressed in reticent or inhibited behavior (e.g., shyness) and evokes thoughts and feelings in others that bring about social connection or caregiving (Keltner et al., 1997). We posited that urban Black American children, given their membership in a longstanding marginalized sub-population of the U.S., are especially likely to evidence shyness as a form of appeasement in navigating their complex social worlds. The study findings largely substantiated this view.

Before discussing evidence regarding the primary study hypotheses, the observed gender difference in levels of perceived shyness first warrants attention. Consistently across grade levels, Black girls were nominated as shy by peers more frequently than were Black boys. This finding aligns with a meta-analysis of shyness research indicating that most studies have found no clear gender differences in shyness prevalence rates, with a small minority of studies finding higher rates among girls and no studies finding higher rates among boys (Else-Quest et al., 2006). It should be noted that our findings speak to gender differences in the prevalence of Black children’s shyness, but they do not speak to potential gender differences in the *expression* of shyness or to the long-term *consequences* of their shyness in terms of academic achievement, socioemotional functioning, or health-related behaviors. As other scholars have noted, the life consequences of shyness depend upon how a culture defines gender roles and ascribes meaning to shy behavior (Chen, 2010; Kerr et al., 1996). Whether the long-term developmental trajectories of shy Black boys and girls differ across the lifespan deserves scholarly attention.

### 5.1. Eliciting Conditions of Black Children’s Shyness

The findings of this study provided at least partial support for the two hypotheses concerning the eliciting conditions of urban Black children’s shyness. In partial support of the *shyness as protection from environmental risk hypothesis* (*H*_1_), Black girls (but not boys) and older (but not younger) children who resided in disadvantaged neighborhoods were more frequently perceived by their peers as being shy. Although relatively few studies have examined links between shyness and SES, shyness also has been found to be more elevated in students coming from lower (vs. higher) socioeconomic backgrounds among adolescents in Pakistan (Shah & Samad, 2025), the United Kingdom (Lawrence & Bennett, 1992), and Saudi Arabia (Asi, 2016). Gender and age were not significant moderators of the shyness–SES association in these studies, so it is unclear why the link between shyness and neighborhood disadvantage was evidenced only among Black girls and older children in our study. Two explanations seem plausible: First, as a whole-body response to threat signals, shyness may cause Black children residing in disadvantaged neighborhoods to “slow down” their mental and emotional processing of environmental cues and their options to avoid them (Andrews, 2020); for reasons not immediately clear, Black girls and older Black children may be more inclined to exhibit this behavioral pattern than are Black boys and younger Black children. Second, lower-SES families tend to socialize their children to develop “hard interdependence”: to be self-protective, to value deference to authority and following the rules, and to have self-restrained behavior at school (Stephens et al., 2014). Black girls and Black early adolescents who display these behavioral patterns may be misinterpreted by peers as being shy. These are speculations that await empirical scrutiny.

The results provided robust support for the *shyness as anticipatory appeasement hypothesis* (*H*_2_). Having low popularity was associated with higher levels of perceived shyness among students in the upper grades but not the lower grades. This finding is consistent with the notion that shyness increasingly functions as a form of appeasement as Black children transition from childhood to early adolescence, though the precise nature of this change cannot be discerned from our cross-sectional data. Nor is it clear from our data whether children who have different types of shyness (e.g., anxious shyness vs. regulated shyness) follow similar or different trajectories in the ascendance of shyness as appeasement. What can be discerned is that compared to residing in disadvantaged neighborhoods, having low popularity was a more powerful predictor of shyness; this is further evidence that proximal (vs. distal) social settings have disproportionate influence on Black children’s behavior and socioemotional functioning (Spencer, 2006).

### 5.2. Hedonic and Care-Eliciting Effects of Shyness

The findings also provided at least partial support for the study hypotheses concerning the hedonic and care-eliciting effects of Black children’s shyness. Models with teacher-rated measures corroborated models with peer nomination measures.

The models provided robust support for the *hedonic effects of shyness hypothesis* (*H*_3_). Among students in the upper grades (but not the lower grades), being widely perceived as shy was associated with higher levels of both peer and teacher measures of prosocial behavior. This pattern is less consistent with patterns shown by children from individualistic cultures and more consistent with patterns shown by children in collectivistic cultures (Chen, 2010). Interestingly, shyness was not positively associated with being well liked by peers (as is typically found in communalistic cultures) nor was it negatively associated with being well liked by peers (as is typically found in individualistic cultures). Rather, the null shyness–acceptance association found here bears closer resemblance to findings with children from “blended” cultures such as Asian Canadian children (Chen & Tse, 2008). However, a notable caveat bears mentioning. Whereas most research examines the simple correlations between shyness and other measures of social adjustment, our analysis controlled for perceived popularity to isolate the unique association between shyness and peer acceptance (and prosocial behavior) above and beyond factors that elicit shyness itself. Notwithstanding this caveat, the null shyness–acceptance association implies that many shy Black youth are well liked, and that shy Black youth as a whole are liked just as much as their non-shy peers. More importantly, given shy children’s self-effacing nature, being liked by just a few peers may be sufficient (or even preferred) for many shy Black children. In-depth interviews with shy Black children can provide insight into the social lives shy Black children want for themselves, and how they feel about the social lives they actually have.

Lastly, the models also provided support for the *care-eliciting effects of shyness hypothesis* (*H*_4_). Being widely perceived as shy was associated with having a closer relationship with the teacher, and this association was stronger among students in the upper grades than among students in the lower grades. Respect for elders is a core value in traditional Black American culture (Wylie, 1971), as is a reliance upon fictive kin and natural mentors (Stern et al., 2023). Our findings suggest that shy Black youth may be especially inclined to develop positive relationships with teachers, coaches, and other natural mentors at school. Given the reciprocal nature of these relationships, teachers may also have a particular fondness for shy Black youth. These findings invite in-depth qualitative research into the nature of the relationships between shy Black youth and their teachers.

### 5.3. Implications for Teachers and Scholars

The present study has clear implications for teachers, yet these messages are in danger of being obscured by louder narratives. Contrary to images of the *shy* child, dominant public narratives (Metzler et al., 2021; Farmer, 2010) and pervasive stereotypes (Opara et al., 2022; Pickett et al., 2014) portray Black children as loud, hostile, and prone to criminality. This trend predisposes Black youth, relative to their White peers, to be viewed as less innocent, more adult-like, and less in need of adult protection (Dumas & Nelson, 2016; Epstein et al., 2017; Ferguson, 2000). In response to such misguided notions, scholars have long called for adults to recognize the fullness of Black students’ humanity: their needs for intimacy and companionship, their yearnings for authentic connections with trusted adults, and the wide breadth of their emotional experience (Legette et al., 2022). The simple act of recognizing shyness as a normative feature of Black children’s socioemotional landscape is an important step toward honoring the fullness of their humanity.

Indeed, stark vulnerability is a fact of life for many urban Black children. When writing about young adolescent Black boys’ visceral feelings of vulnerability, clinical psychologist Howard Stevenson noted, “[Many Black boys] feel ‘missed’ in the sense of feeling misrepresented and misinterpreted, ‘dissed’ in the sense of feeling disrespected and distorted, and ‘pissed’ in the sense of feeling intense anger related to the devaluation experienced since early childhood from societal, familial, and interpersonal rejection” (Stevenson, 2004, p. 61). Related to this theme, a second implication for educators is to resist misinterpreting Black children’s shyness as aloofness, lack of interest, or academic disengagement, but instead to see their shyness as an attempt to hide from plain view any number of anxieties: fear of giving the wrong answer, a belief that asking for help is a sign of immaturity, or frustration with the ambiguity of an assignment. Given the complexity of feelings that often accompany shyness, having genuine empathy for shy Black children may present opportunities for natural mentorship—perhaps more so than for shy children of other ethnic–racial backgrounds. Evidence from predominantly middle-class White samples indicates that shy children enjoy many benefits from having high-quality relationships with their teachers yet are less likely to develop such relationships as they progress through schooling (Crozier, 2020). Present study findings revealed a different pattern among Black students, as shy early adolescents were *more likely* than younger shy children to have close relationships with their teachers. This is a positive sign for teachers who wish to provide a safe haven for Black students who are learning to navigate the many challenges that adolescence presents.

Beyond these two suggestions, the study implications for teachers are limited. As an exploratory study into Black children’s shyness, the present study focused on variables specifically germane to the presumed biologically prepared function of shyness: *why* it is that some Black children are shy at school. A comprehensive analysis of shy Black children’s school adjustment—*how* they fare socially, emotionally, and academically—was beyond the scope of this analysis. To be of greater utility to educators, future research on shy Black children should include longitudinal measures of their ethnic–racial identity and socialization, peer relationships (e.g., friendships, peer rejection), perceptions of the classroom environment, academic functioning, internalizing symptomology (e.g., depression), and global assessments of school adjustment (e.g., absenteeism). Developing multiple lines of inquiry on shy Black youth is clearly needed to better inform educators and clinical professionals. Given the dearth of research on this population, qualitative research that centers shy Black youth’s own voices may be the best place to start.

### 5.4. Study Limitations and Conclusions

A few study limitations deserve consideration. First, the non-randomized, cross-sectional study design precludes inferences about causality. This study’s conceptual framework posited that situations presenting danger or lacking safety (i.e., eliciting conditions) give rise to shyness as an appeasement strategy, which in turn gives rise to safety-promoting hedonic and care-eliciting behaviors. It may be the case, however, that in the long run shyness is a stronger predictor of low status than vice-versa. Longitudinal studies are needed to clarify the directionality of effects. Second, the study lacked important life-history information regarding early life adversity (see Poole et al., 2020), stability of household membership, parent–child relationship quality, and other factors known to influence Black children’s socioemotional development (Barbarin et al., 2019). Likewise, we were unable to distinguish between children who developed shyness early in life and children who developed shyness later in childhood or early adolescence. Given that early-onset shyness stems from a behaviorally inhibited temperament (Kagan et al., 1988) and later-onset shyness stems from the increasing salience of social comparisons (Crozier, 2000), early- and late-onset shyness may evidence different temporal patterns of appeasement-related behaviors. These concerns lie beyond the scope of this report.

Offsetting the above-described limitations were several notable strengths. We used multiple sources of information (peers, teachers, and census data) to examine shyness in a child population that previously had never been studied. We integrated evolutionary and cultural perspectives on human shyness to postulate ecologically valid hypotheses concerning the function of shyness in two sequential stages of development. We used sociometric procedures to inquire into how Black American children identify and make meaning of shyness within their peer group cultures, thereby enhancing ecological validity (Chen, 2010). Based on these strengths, our findings reveal a striking age-related pattern for urban Black American children: though shyness is a normative experience for Black children of all ages, it is in early adolescence that shyness begins to function as appeasement and facilitate Black children’s prosocial affiliations with peers and teachers.

## Figures and Tables

**Table 1:** Study participants.

	No. of Participants			Age in Years			
	Total	Boys	Girls	*M*	*SD*	Min	Max
Grade 3	99	55 (56%)	44 (44%)	9.4	0.57	8.6	11.6
Grade 4	106	37 (35%)	69 (65%)	10.3	0.53	9.6	12.3
Grade 5	121	59 (49%)	62 (51%)	11.2	0.49	10.4	12.6
Grade 6	119	53 (46%)	66 (54%)	12.3	0.51	11.2	13.7
Grades 3 and 4	205	92 (45%)	113 (55%)	9.9	0.71	8.6	12.3
Grades 5 and 6	240	112 (47%)	128 (53%)	11.7	0.74	10.4	13.7
Total	445	204 (46%)	241 (54%)	10.9	1.21	8.6	13.7

*Note*: In the study analyses, grade cohort was operationalized dichotomously: a younger cohort (grades 3–4) versus an older cohort (grades 5–6).

**Table 2:** Bivariate correlations and descriptive statistics for study variables.

	1	2	3	4	5	6	7	8	9	Raw Scores	
										*M*	*SD*
1. Neighborhood disadvantage	--									0	1
2. PN Shyness	0.06	--								0.09	0.12
3. PN Popularity	0.00	−0.20 **	--							0.26	0.22
4. PN Prosocial behavior	−0.04	0.15 **	0.36 **	--						0.27	0.15
5. PN Likeability	0.03	−0.12 **	0.75 **	0.54 **	--					0.28	0.16
6. PN Gets along with teacher	−0.01	0.26 **	0.27 **	0.77 **	0.42 **	--				0.34	0.18
7. TR Prosocial behavior	−0.06	0.10 *	0.02	0.46 **	0.17 **	0.46 **	--			3.56	0.95
8. TR Disruptive behavior	0.02	−0.27 **	0.06	−0.47 **	−0.08	−0.51 **	−0.73 **	--		2.59	1.07
9. TR Student–teacher closeness	−0.06	0.15 **	−0.07	0.37 **	0.06	0.38 **	0.69 **	−0.71 **	--	3.93	0.88

*Note*: PN = peer nomination measure. TR = teacher-reported measure. ** *p* < 0.01; * *p* < 0.05.

**Table 3:** Multilevel models of prosocial behavior and likeability on shyness.

	PN Prosocial Behavior		TR Prosocial Behavior		PN Likeability	
	*b*	95% CI of *b*	*b*	95% CI of *b*	*b*	95% CI of *b*
Model 1						
Gender	0.038 **	[0.025, 0.050]	0.153 **	[0.064, 0.241]	0.012 *	[0.002, 0.022]
Cohort	0.001	[−0.028, 0.031]	0.015	[−0.191, 0.221]	0.013	[−0.017, 0.043]
Neighb. disadv.	−0.010	[−0.024, 0.004]	−0.064	[−0.158, 0.030]	0.004	[−0.007, 0.015]
Popularity	0.047 **	[0.034, 0.059]	−0.003	[−0.088, 0.081]	0.110 **	[0.101, 0.120]
Shyness	0.023 **	[0.010, 0.035]	0.048	[−0.037, 0.134]	0.001	[−0.009, 0.011]
Model 2						
Gender × shyness	−0.001	[−0.014, 0.011]	−0.082 ^†^	[−0.170, 0.006]	−0.008	[−0.018, 0.003]
Cohort × shyness	0.019 **	[0.007, 0.031]	0.113 **	[0.032, 0.195]	0.007	[−0.003, 0.016]

*Note*: PN = peer nomination measure. TR = teacher-reported measure. ** *p* < 0.01; * *p* < 0.05. ^†^ *p* < 0.10.

**Table 4:** Multilevel models of student–teacher closeness measures on shyness.

	PN Gets Along with Teacher		TR Student–Teacher Closeness	
	*b*	95% CI of *b*	*b*	95% CI of *b*
Model 1				
Gender	0.032 **	[0.016, 0.047]	0.056	[−0.021, 0.132]
Cohort	0.019	[−0.026, 0.064]	0.006	[−0.236, 0.247]
Neighborhood disadv.	−0.007	[−0.023, 0.009]	−0.057	[−0.139, 0.025]
Popularity	0.045 **	[0.031, 0.060]	−0.046	[−0.119, 0.027]
Shyness	0.041 **	[0.027, 0.055]	0.099 **	[0.026, 0.172]
Model 2				
Gender × shyness	−0.007	[−0.022, 0.008]	−0.080 *	[−0.156, −0.004]
Cohort × shyness	0.017 *	[0.003, 0.031]	0.064	[−0.006, 0.135]

*Note*: PN = peer nomination measure. TR = teacher-reported measure. ** *p* < 0.01; * *p* < 0.05.

## Data Availability

The data presented in this study are available upon request from the corresponding author.

## Note

1 Entering the search terms “Black, shyness” (or “Black, shy”) into Google Scholar as title words results in six matching articles: three articles concern shyness in Black American adults, two articles concern shyness in the black rat, and one article concerns shyness in black widow spiders. Entering the search terms “African American, shyness” or “African American, shy” results in zero matches.
